# Iodine contrast agents do not influence Platelet-Rich Plasma function at an early time point in vitro

**DOI:** 10.1186/s40634-018-0162-4

**Published:** 2018-10-29

**Authors:** B. Dallaudiere, A. Crombé, A. P. Gadeau, L. Pesquer, A. Peuchant, C. James, A. Silvestre

**Affiliations:** 1 Interventional Musculoskeletal Radiology Department, Clinique du sport de Bordeaux, F-33700 Mérignac, France; 2 Centre de Résonance Magnétique des Systèmes Biologiques, UMR5536, CNRS, F-33000 Bordeaux, France; 3 Université de Bordeaux, F-33076 Bordeaux, France; 4 Department of Radiology, Institut Bergonié, F-33000 Bordeaux, France; 5 Biology of Cardiovascular Diseases, INSERM U1034, F-33600 Pessa, France; 6 Department of Pathology, Clinique du sport de Bordeaux, F-33700 Mérignac, France

**Keywords:** Platelet, Arthritis, Iodine contrast agent, Intra-articular infiltration

## Abstract

**Background:**

Iodine contrast agents (ICAs) are routinely used by radiologists to help guide intra-articular infiltrations. The aim of this study was to assess the in vitro effects of ICA on platelet function of human autologous Platelet-Rich Plasma (PRP).

**Methods:**

One hundred thirty-seven consecutive patients with symptomatic femoral-patellar osteoarthritis were included. All were addressed to our institution for a fluoroscopy-guided intra-articular PRP infiltration of the pathological femoral-patellar joint. For each patient, 500 μl of PRP were sampled before intra-articular injection. First, PRP samples were mixed with 50 μl of 2 widely used ICA: Visipaque270® (Iodixanol, *n* = 58) and Iopamiron200® (Iopamidol, *n* = 69). PRP concentration ([PRP]) was measured at different delays of incubation (*t* = 0, 5, 10, 15, 20 and 30 min) enabling to calculate PRP ratio (defined as [PRP](t)/[PRP](0mn)) at each delay, for each mixture, in order to quantitatively assess the influence of ICA on PRP ratio. Second, the PRP samples of 10 additional patients were mixed with Visipaque270®, Visipaque270®, Iopamiron200® and phosphate buffer saline (PBS: control solution) in order to qualitatively assess the influence of ICA on platelet aggregation, using ADP, Collagen, Arachidonic acid and TRAP tests. The surface expression of human P-selectin, a marker of α-granule release, in the PRP + Visipaque270® and PRP + Iopamiron200® mixtures was finally compared. Repeated-measures ANOVA, classical 2-way ANOVA and Wilcoxon matched-pairs test were used to study the influence of ICA on PRP quality.

**Results:**

There was no significant change in PRP ratio during the first 30mn of incubation (*p* = 0.991) whatever the ICA (*p* = 0.926). Whatever the aggregation test, there was no significant difference in the percentage of platelet aggregation between PRP + PBS, PRP + Visipaque270® and PRP + Iopamiron200® (*p* = 0.998), nor between PRP + PBS and PRP + Visipaque320® (*p* = 0.470). Finally, there was no significant difference in P-selectin expression between the PRP + Visipaque270® and PRP + Iopamiron200® mixtures (*p* = 0.500).

**Conclusion:**

At early delays of incubation, Visipaque® and Iopamiron®, which are two widely used ICA for intra-articular infiltrations, did not influence the in vitro platelet function nor the quality of PRP.

## Background

Osteoarthritis (OA) is a chronic degenerative joint condition characterized by a progressive destruction of the articular cartilage leading to pain and functional loss (Cross et al. 2014). OA affects more than 39 million people in Europe. These changes lead to a degradation of the extracellular matrix and a reduction of tissue cellularity. Furthermore, due to the little proliferative possibility of articular chondrocytes, cartilage has a limited self-repair ability. Consequently, even minor injuries may progress to important joint degenerations (Laadhar et al. 2007). Small lesions are generally repaired by migration of chondrocytes, while larger lesions are repaired by the formation of a fibrocartilage with impaired biomechanical properties Fernandes et al. (2002). Recent repair treatments have been explored, including microfracturing or osteochondral allograft transplantation, but they are invasive, with variable prognosis (Gomoll et al. 2012). Currently, oral nonsteroidal anti-inflammatory drugs (NSAID), intra-articular corticosteroids or hyaluronic acid are widely used to relieve OA. Unfortunately, these treatments do not have curative effects on the inflammatory process of OA (Nelson et al. 2014). To address this question, new repair strategies have been developed. Platelet-rich plasma (PRP) is a plasma with 3 to 8 times higher platelet concentration than in the blood, which enables higher concentrations of active growth factors such as platelet-derived growth factors, transforming growth factors β and vascular endothelial growth factor. PRP might promote stem cells recruitment and fibroblast collagen production. Studies in human and animal models suggest that PRP would improve cartilage repair (Sakata et al. 2015). In a recent meta-analysis of 10 level I randomized controlled trials, intra-articular PRP injection was found to better relieve pain and to improve function scores at 1 year post-injection compared to saline and hyaluronic acid (Dai et al. 2017). However, contradictory results have been published (Mascarenhas et al. 2015) but these studies were performed with different types of PRP on different joints and included small heterogeneous populations from first to late stages of OA. Notably, the techniques for intra-articular PRP infiltration were not standardized: some were clinically-guided, others imaging-guided. Radiologists are used to adding iodine contrast agent (ICA) to the injected substance (for instance corticosteroids or hyaluronic acid) to improve the precision of fluoroscopy-guided intra-articular infiltrations. Opacification of the symptomatic joint by ICA confirms that the needle is correctly positioned. By analogy, radiologists have come to mix PRP and ICA during the imaging-guided procedures in order to ascertain that PRP is correctly infiltrated. However, the effect of ICA on platelets is debated and may depend on the type of ICA Aspelin et al. (2006). In practical applications, knowing that the quality of PRP is not altered by ICA would encourage to the use of ICA to better guide the needle. Conversely, knowing that ICA could alter PRP would discourage the use of ICA during PRP-related procedure. Thus, the aim of this study was to investigate the in vitro effect of ICA on platelet concentration, activation and degranulation from autologous PRP.

## Methods

The institutional ethical committee approved the study. Signed informed consent was obtained for all patients who participated*.*

### Patients

From May 2016 to January 2017, a total of 137 consecutive patients were prospectively enrolled in the study. Inclusion criteria were: age (< 18 years old), radiographically documented OA in the patellofemoral compartment of the symptomatic knee (grade 2 and 3 according to the Kellgren-Lawrence classification), diagnosis of primary OA confirmed at least 3 months prior to the study, failure of non-operative treatment performed at least 6 weeks prior to the study (rehabilitation using analgesic physiotherapy and eccentric work), indication for intra-articular PRP injection validated by a sports medicine physician or an orthopaedic surgeon, infiltration performed at our musculo-skeletal interventional radiology institution. Exclusion criteria were: pregnancy, infections, previous corticosteroid treatment, platelet dysfunction and immunodeficiency. None of the 137 patients met the exclusion criteria. The study included 78 men and 59 women (median age: 45-years old, range 28–64).

### PRP preparation

Patients were referred to a clinical pathologist. For each patient, 22 mL of venous blood was collected in a syringe containing 1.5 mL of citrate anticoagulant. Blood was centrifuged at 570 g for 8 min and a final volume of 3.5 mL of PRP was recovered in the lower plasma layer. The platelet concentration factor within the final volume was 2.5, as verified by platelets counting under an automated hematology cell analyzer (ABX Pentra 60, Horiba, Kyoto, Japan). Platelets and leucocytes amounts were controlled within each PRP sample Dallaudière et al. (2014). Finally, 500 μl of autologous PRP per patient were sampled prior to intra-articular PRP injection.

### In vitro quantitative assessment

After anonymization of the PRP samples, 500 μl of PRP were randomly mixed with 50 μl of different ICA (PRP + ICA): Iodixanol 270 mg I/mL (Visipaque270®, GE Healthcare, ChICAgo, Illinois, USA) (*n* = 58) or Iopamidol 200 mg I/mL (Iopamiron200®, Bracco imaging, Milan, Italy) (*n* = 69). Visipaque270® is a non-ionic, hexa-iodinated solution (iodixanol) with an osmolality of 290 mOsm/kg whose excipients are trometanol, sodium chlorure, calcium chlorure, sodium edetate calcium, water and chlorohydrin acid. Iopamiron200® is a non-ionic, tri-iodinated solution with an osmolality of 413 mOsm/kg whose excipients are trometanol, sodium hydroxyd, sodium edetate calcium, water and chlorohydrin acid.

The platelet concentration (G/L) within PRP alone (*t* = 0mn) and PRP + ICA at several delays of incubation was quantified (namely, 5, 10, 15, 20, 30mn). ‘Thirty minutes’ was arbitrarily chosen as the last point in time because PRP is always infiltrated within this delay at our institution and because of lack of enough PRP after this delay for statistical comparisons. Additionally, in vivo*,* it is recommended to acquire X-rays within the 30mn following the infiltration of ICA because the concentration in ICA continuously decreases as it is likely absorbed by the synovia (Obermann et al. 1989; Omoumi et al. 2009). Thus, the interaction between PRP and ICA should also decrease after this incubation delay. Then, the pathologist counted the number of platelets within each sample at each delay of incubation (with an automated cell analyser) and analysed the morphology of the platelets (with an optical microscope with a magnification of 40) blinded to the clinical data. PRP ratio was defined as the concentration of PRP within the PRP + ICA mixture at a given time point divided by the initial concentration of PRP (PRP ratio = [PRP](t)/[PRP](0mn)).

### In vitro qualitative assessment

First, our objective was to compare the platelet function between two different ICAs (Visipaque270®, Iopamiron200®) and a control solution (phosphate buffer solution, PBS). Five patients were included in this part of the study. Their PRP samples were divided in three equal sub-samples and mixed with 50 μL of PBS, Visipaque270® and Iopamiron200®. For each mixture (PRP + PBS, PRP + Visipaque270®, PRP + Iopamiron200®), the platelet aggregation was evaluated according to 4 tests after a delay of incubation of 30mn, using: (i) 10 μmol/L adenosine diphosphate (ADP, Sigma Aldrich Chimie, Lyon, France), (ii) 1 mmol/L arachidonic acid (AA, Nu-Chek-Prep, Elysian, Minnesota, USA), (iii) 25 μmol/L thrombin receptor activating peptide (TRAP, NeosystemSA, Strasbourg, France), and (iv) 2 μg/μL of equine tendon collagen (Horm-Chemie collagen, Nycomed Pharma, Munich, Germany). The tests were achieved in an aggregometer (APACT® 4004, ELITechGroup, Salon de Provence, France) according to standard procedures and during an aggregation test time of 300 s.

Second, we investigated whether a different concentration in Iode for the same molecule of ICA could change the platelet function. To do so, we compared the platelet function of two mixtures: PRP + PBS and PRP + Visipaque320® (: iodixanol 320 mg I/mL, instead of 270 mg I/mL), with the same aggregation tests on five other patients.

Third, cell surface expressions of P-selectin, which is a marker of alpha-granule release, in Visipaque270® and Iopamiron200® mixtures were compared. The measures were performed on the PRP samples of 5 additional patients before and after addition of ICA, using VH10, which is a murine monoclonal antibody produced by our group Cattaneo et al. (2013). The mean fluorescence intensity of P-selectin was measured on resting platelets and after stimulation with 25 μM TRAP, enabling to calculate the P-selectin expression ratio (defined as the ratio of mean fluorescence intensity of P-selectin after TRAP stimulation and before TRAP stimulation).

### Statistical analyses

Gaussian distribution was tested with the Shapiro-Wilk normality test. The initial PRP concentrations (incubation delay *t* = 0mn) in the presence of different ICA were compared using the Mann-Whitney test. The influences of both ICA and the delay of incubation on PRP ratio were evaluated with a 2-way ANOVA (with post-hoc Tukey test for multiple comparisons). The influences of ICA and the aggregation tests on the percentage of platelet aggregation were evaluated with a repeated-measures 2-way ANOVA (with a post-hoc Sidak test for multiple comparisons). The P-selectin ratios between the PRP + Visipaque270® and PRP + Iopamiron® mixtures were compared using a non-parametric Wilcoxon matched-pairs signed rank test.

Data are presented as mean ± standard deviation. A *p*-value of less than 0.05 was deemed significant. Statistical analyses were performed using SPSS statistical software (version 21.0, Chicago, Illinois, USA) and Graphpad Prism (GraphPad Software, Inc., version 7, La Jolla, California, USA).

## Results

### In vitro quantitative assessment

**Fig. 1:**
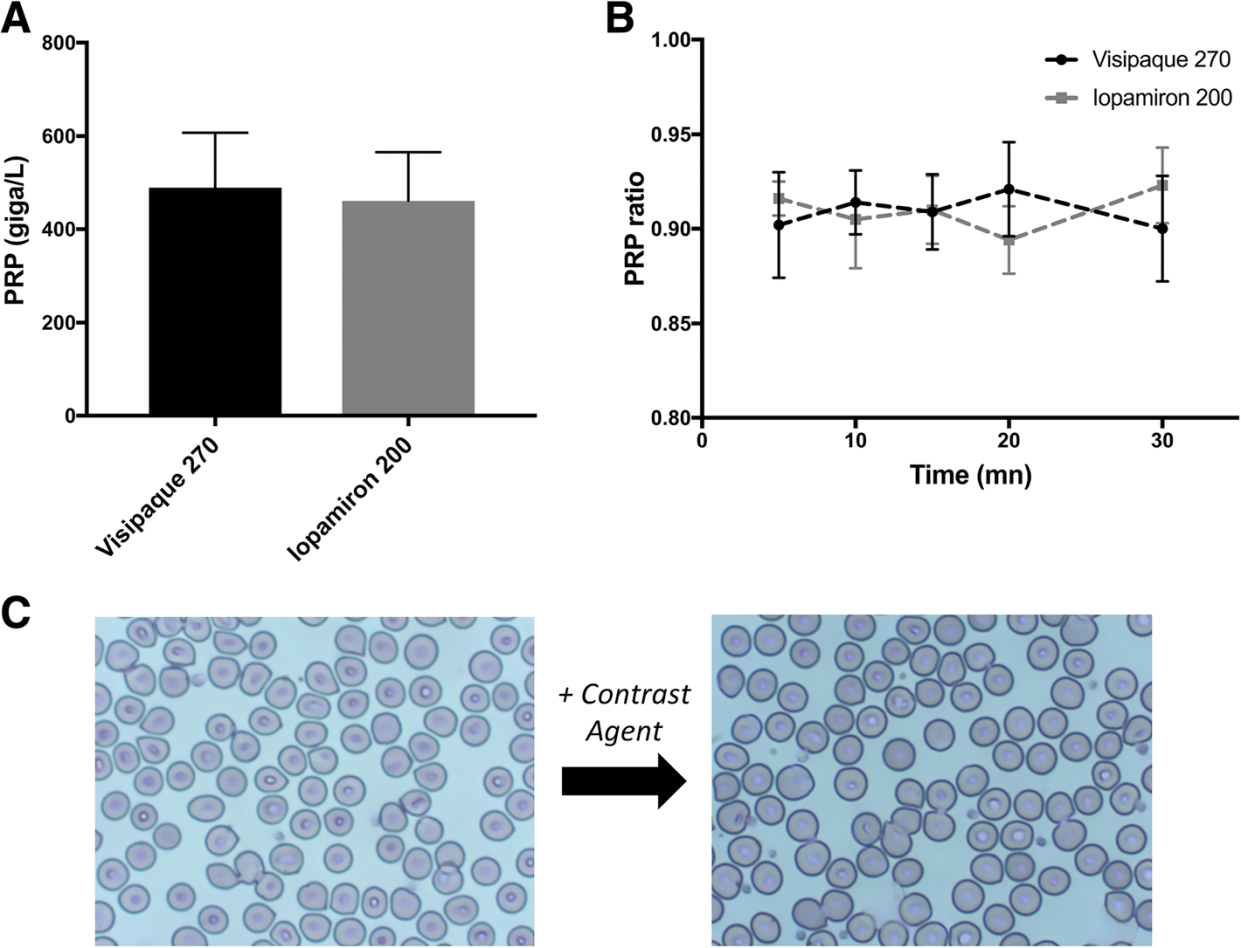
In Vitro quantitative assessment. **a**Initial PRP concentration in the samples (*t* = 0), in the presence of Visipaque270® and Iopamiron200®. **b** Evolution of PRP ratio as a function of time (5 incubation delays: 5, 10, 15, 20, 30mn), for each contrast agent group (Visipaque270® and Iopamiron200®). **c** Morphological aspect of platelets before and after exposure to iodine contrast agent: no change was seen

Initially (*t* = 0mn), there was no significant difference in [PRP] between the following mixtures: PRP + Visipaque270® and PRP + Iopamiron200® mixtures (489.3 +/− 118 G/L versus 460.9 +/− 104.7 G/L, respectively, *p* = 0.250) (Fig. 1a).

A 2-way ANOVA was conducted to examine the effect of ICA, the incubation delay (from 5 to 30mn) and their interaction on PRP ratio (Fig. 1b). There was no significant influence of the incubation delay on PRP ratio (F(4,128) = 0.069, *p* = 0.991), neither was there a significant influence of the ICA (F(1,128) = 0.009, *p* = 0.926), nor a significant influence of their interaction on PRP ratio (F(4,128) = 2.654, *p* = 0.057). Table 1 shows the values of PRP ratio as a function of the incubation delay.

**Table 1:** PRP ratio in PRP + Visipaque270® and PRP + Iopamiron200® at different incubation delays

Incubation Delay (mn)	PRP ratio	
	Visipaque270® (n = 58)	Iopamiron200® (n = 69)
5	0.902 ± 0.028	0.916 ± 0.009
10	0.814 ± 0.014	0.905 ± 0.026
15	0.909 ± 0.020	0.910 ± 0.018
20	0.921 ± 0.025	0.894 ± 0.018
30	0.900 ± 0.028	0.923 ± 0.020

NOTE: PRP ratio is defined as the concentration in PRP in the mixture at each incubation delay divided by the initial concentration in PRP. Results are mean ± sd

The optical microscope examination at the end of the incubation delay (30mn) did not demonstrate morphological change of the platelets for both ICA (Fig. 1c).

### In vitro qualitative assessment

First, the percentage of platelet aggregation within the different mixtures (PRP + PBS, PRP + Visipaque270® and PRP + Iopamiron200®) was evaluated after an incubation of 30mn in the presence of ADP, Collagen, AA and TRAP (: 4 tests of aggregation).

**Fig. 2:**
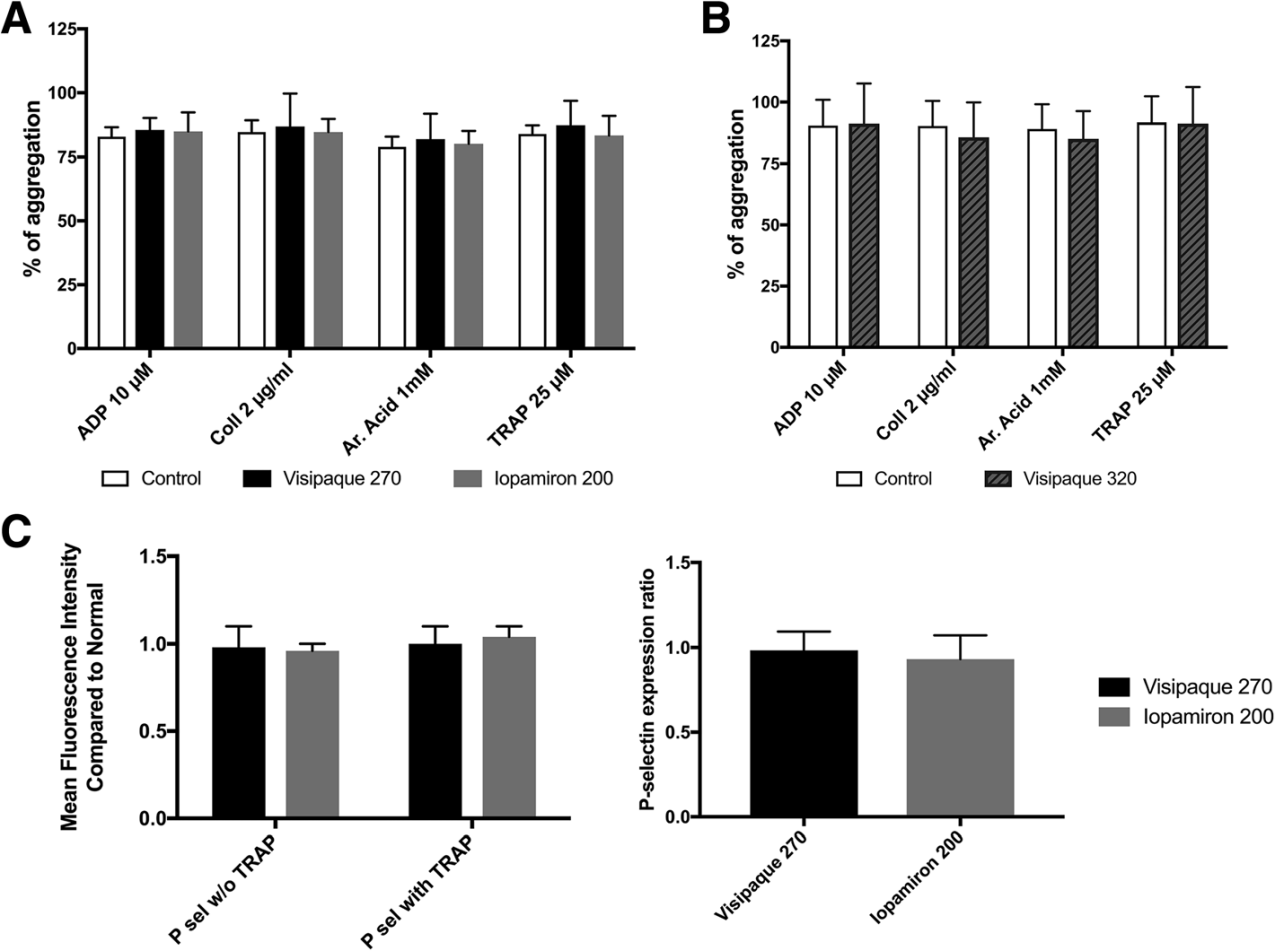
In Vitro qualitative assessment. **a** Platelet aggregation depending on the iodine contrast agent. Three groups were compared: control (PRP + phosphate buffer solution), Visipaque270® (PRP + Visipaque270®) and Iopamiron200® (PRP+ Iopamiron200®) (**b**) Platelet aggregation depending on the iodine concentration of Visipaque®. Two groups were compared: control (PRP + phosphate buffer solution) and Visipaque320®. The percentage of platelet aggregation was assessed by 4 tests: in the presence of adenosine diphosphate (ADP), Collagen, Arachidonic acid (Ar. acid) and thrombin receptor activating peptide (TRAP). **c** Degranulation: Surface expression of human P-selectin in the presence of PRP + Visipaque270® or PRP + Iopamiron200®, before and after exposure to 25 μM of TRAP. P selectin expression is expressed as the mean fluorescence intensity compare to normal. P-selectin expression ratio corresponds to the ratio between expression after TRAP exposure and before TRAP exposure

The solution that was added to PRP did not have a significant effect on the percentage of platelet aggregation (F(2,5) = 0.907, *p* = 0.414), neither did the interaction between the aggregation test and the solution added to PRP (F(6,5) = 0.064, *p* = 0.998) (Fig. 2a).

When another iodine concentration of Visipaque® was tested (PRP + Visipaque320® with 320 mg I/mL versus PRP + PBS) using the 4 same tests of aggregation, the solution that was added to PRP did not have a significant effect on the percentage of aggregation (F(1,5) = 2.110, *p* = 0.166), neither did the interaction between the aggregation test and the added solution (F(3,5) = 0.885, *p* = 0.470) (Fig. 2b). Post-hoc tests are given in Table 2.

**Table 2:** Comparisons in percentage of aggregation between different mixtures, assessed by four aggregation tests

Comparisons	Aggregation tests			
	ADP	Collagen	AA	TRAP
PRP + Iopamiron200® vs. PRP + Visipaque270®	> 0.999	0.794	0.940	0.475
PRP + PBS vs. PRP + Visipaque270®	0.839	0.641	0.720	0.895
PRP + PBS vs. PRP + Iopamiron200®	0.817	0.993	0.963	0.748
PRP + PBS vs PRP + Visipaque320®	0.993	0.876	0.761	> 0.999

NOTE. Results are the p-value of the post-hoc tests of the 2-way ANOVA

The different mixtures were: PRP + Iopamiron200**®**, PRP + Visipaque270**®**, PRP + Visipaque320**®**, PRP+ PBS**®**

Abbreviations: *AA* arachidonic acid, *ADP* adenosine diphosphate, *PBS* phosphate buffer solution, *TRAP* thrombin recepto activating peptide, *vs*. versus

Given the ability of the platelets to release alpha-granule, the mean fluorescence intensity of P-selectin was not significantly modified after adding TRAP; neither was it in the presence of Visipaque270® (*p* > 0.999) nor in the presence of Iopamiron200® (*p* = 0.500). The ratio of P-selectin expression after adding TRAP and ICA was not significantly different between Iopamiron200® and Visipaque270® (0.931 +/− 0.141 versus 0.984 +/− 0.111, p = 0.500) (Fig. 2c).

## Discussion

In this study, we investigated the potential influence of ICA on platelet function through extensive in vitro analyses. We demonstrated that the concentration in PRP was not altered in the presence of ICA during a delay of incubation of 30mn. We did not observe an alteration of the platelet function in the presence of ICA according to several aggregation tests. The ability of the platelets to release alpha-granule was not modified in the presence of ICA. Altogether, our results suggest that platelets from PRP samples were not quantitatively and functionally modified by adding ICA.

The technique for intra-articular infiltration with PRP lacks standardization when it comes to the platelet concentration in PRP samples, the volume of PRP to infiltrate, the optimal moment for an injection of PRP in the course of OA, the number of injections to perform and the adjunction of NSAIDs and/or anaesthetics and/or ICA. All these technical points could also be optimized for each joint (Campbell et al. 2015). In our study, we standardized the collection system with a single centrifugation step and we obtained autologous PRP samples with a 2.5–3 times higher concentration of platelets, as recommended in the literature Dallaudière et al. (2014). Optimizing the chance to correctly target the pathological joint requires guidance by fluoroscopy and validation of the right position of the needle by opacification with ICA. For such a controversial treatment as PRP, we believe that it is crucial to demonstrate that the substance was well delivered in the joint and not in adjacent tissue whether clinical trials or daily routine. The efficacy of PRP may have been ill estimated in previous studies without the use of ICA because the PRP may have been delivered outside of the pathological joint. Radiologists are used to mixing ICA and hyaluronic acid, or ICA and corticosteroids. By analogy, they routinely mix ICA and PRP. Yet, the interaction between ICA and PRP can be questioned. Prior reports suggest that ICA may have an impact on platelet function. In a study that investigated the adverse events of several ICA based on 48,261 reports, the authors highlighted the frequency of bleeding and clotting disorders likely due to alterations in platelet function (Seong et al. 2013). Cavalli et al. showed that prostaglandin E2, which can modify platelet function, was released in the synovial fluid of patients following a knee arthrography with iothalamate and iopamidol, which may explain the occurrence of transient arthritis (Cavalli et al. 1987). In vitro studies showed that platelet activation was not affected by non-ionic ICA (Li et al. 1997), but may modify platelet aggregation and degranulation (Heptinstall et al. 1998). However, these studies should be carefully analysed because of the heterogeneous study design, either on PRP or on blood samples. Herein, our results did not show any in vitro influence of ICA on PRP within a delay of 30mn.

The benefits of PRP depend on the release of bioactive compounds at the optimal moment, through platelets activation and aggregation (Mascarenhas et al. 2015). We excluded patients who were treated by NSAID treatment in order to limit inappropriate platelet activation and aggregation. Adding NSAID to PRP decreased the storage of α-granules and inhibited the activation and aggregation of platelets. In vivo, the function of platelets in autologous PRP in patients who were treated by NSAID is impaired, resulting in a lower quality of PRP bioactive compounds (Schippinger et al. 2015). Moreover, the adjunction of anaesthetics or corticosteroids to intra-tendinous PRP injections demonstrated a significant decrease of tenocytes proliferation and cell viability. These results suggest that adding anaesthetics and/or corticosteroids to PRP would compromise the potential benefits of PRP and the cell viability where the tendon was injured (Carofino et al. 2012). Furthermore, Bausset et al. showed that, in vitro, anaesthetics such as Xylocaine® and Naropin® (belonging to the N-alkyproline anilides group) may compromise PRP potential benefit (Bausset et al. 2014). Herein, the potential interaction between anaesthetics, ICA and PRP was not investigated although anaesthetics are always available during the procedures, at the patient’s request.

In our study, the function of platelets was investigated using aggregation tests and degranulation tests through the measurement of the P-selectin expression (Prüller et al. 2011). The measurement of the P-selectin expression with Flow cytometry is the gold standard method to evaluate α-granule release after platelets stimulation by standardized inductors of platelet activation (Sakata et al. 2015). Bioactive compounds stored in the α-granules such as platelet-derived growth factors or transforming growth factors β, would not have been adequately released if this pathway had been altered by ICA.

Our study has limitations. First, as a pilot study, few patients were included in the in vitro qualitative assessment. The same aggregation tests were performed on 5 patients under 3 conditions (PRP + PBS, PRP + visipaque270®, PRP + iopamiron200®) and on 5 other patients under 2 conditions (PRP + PBS, PRP + visipaque320®). Even if the number of patients was low, the post-hoc tests did not show any tendency towards a difference in the percentage of aggregation under either of the two conditions. The percentages of aggregation (and their standard deviation) under all conditions were very similar to the one under the control condition (: PRP + PBS alone). Second, our study groups consisted in patients with heterogeneous levels of activity, from top-athletes to inactive patients. However, we clearly defined our inclusion criteria and we do not believe that the level of activity could have influenced the interactions between ICA and PRP in an in vitro study. Third, the methods of our in vitro qualitative assessment could be questioned. We did not quantify growth factors and cytokines in the PRP samples before and after ICA adjunction. Furthermore, we used citrate as an anticoagulant instead of hirudin though citrate was suspected to mask pro-aggregatory effects of Iopamiron® in blood samples (Heptinstall et al. 1998). However, this effect was not observed with ‘hirudinized’ PRP samples instead of ‘hirudinized’ blood samples. Fourth, all the in vitro tests were performed within a short delay of incubation (*t* = 30mn). Even if we did not identify an influence of ICA on PRP properties during this delay, one cannot eliminate a potential late interaction after the delay of 30mn. However, it should be noted that the concentration of ICA within the joints rapidly decreases with time. Obermann et al. showed that the best diagnostic quality for knee arthrography was obtained when radiographs were acquired within 23mn (Obermann et al. 1989). After this delay, ICA is absorbed by the synovia and is eliminated through blood circulation. That is why it is commonly recommended to limit the delay between ICA infiltration and image acquisition (Omoumi et al. 2009). Finally, the clinical benefit of adding ICA to PRP compared to PRP alone has not been studied, neither have the clinical effects on patients from our series once PRP and ICA were finally injected in the joint. A randomized controlled trial could be considered to compare ICA-helped, imaging-guided, intra-articular PRP infiltration versus direct intra-articular PRP infiltrations on knee OA following OARSI clinical trial recommendations (McAlindon et al. 2015).

To conclude, our results suggest that platelets from PRP samples were not in vitro quantitatively and qualitatively modified by adding ICA. These results need to be confirmed by in vivo studies with clinical outcome.

## Abbreviations

AA: Arachidonic acid
ADP: Adenosine diphosphate
ICA: Iodine contrast agent
NSAID: Nonsteroidal anti-inflammatory drugs
OA: Osteoarthritis
PBS: Phosphate buffer saline
PRP: Platelet-rich plasma
TRAP: Thrombin receptor activating peptide

## References

[CR1] Aspelin P, Stacul F, Thomsen HS, Morcos SK, van der Molen AJ (2006). Members of the Contrast Media Safety Committee of the European Society of Urogenital Radiology (ESUR) Effects of iodinated contrast media on blood and endothelium. Eur Radiol.

[CR2] Bausset O, Magalon J, Sabatier F (2014). Impact of local anaesthetics and needle calibres used for painless PRP injections on platelet functionality. Muscles Ligaments Tendons J.

[CR3] Campbell KA, Saltzman BM, Cole BJ (2015). Does intra-articular platelet-rich plasma injection provide ClinICAlly superior outcomes compared with other therapies in the treatment of knee osteoarthritis? A systematic review of overlapping meta-analyses. Arthroscopy.

[CR4] Carofino B, Chowaniec DM, Mazzocca AD (2012). Corticosteroids and local anesthetics decrease positive effects of platelet-rich plasma: an in vitro study on human tendon cells. Arthroscopy.

[CR5] Cattaneo M., Cerletti C., Harrison P., Hayward C. P. M., Kenny D., Nugent D., Nurden P., Rao A. K., Schmaier A. H., Watson S. P., Lussana F., Pugliano M. T., Michelson A. D. (2013). Recommendations for the standardization of light transmission aggregometry: a consensus of the working party from the platelet physiology subcommittee of SSC/ISTH. Journal of Thrombosis and Haemostasis.

[CR6] Cavalli F, Mucci MP, Cisternino M (1987). Prostagnaldin E2 liberation in the synovial fluid induced by organo-iodinated contrast media. Interrelations with the genesis of post-arthrographic pain. Radiol Med.

[CR7] Cross M, Smith E, March L (2014). The global burden of hip and knee osteoarthritis: estimates from the global burden of disease 2010 study. Ann Rheum Dis.

[CR8] Dai WL, Zhou AG, Zhang H, Zhang J (2017). Efficacy of platelet-rich plasma in the treatment of knee osteoarthritis: a meta-analysis of randomized controlled trials. Arthroscopy.

[CR9] Dallaudière B, Pesquer L, Serfaty JM (2014). Intratendinous injection of platelet-rich plasma under US guidance to treat tendinopathy: a long-term pilot study. J Vasc Interv Radiol.

[CR10] Fernandes JC, Martel-Pelletier J, Jean-Pierre Pelletier JP (2002). The role of cytokines in osteoarthritis pathophysiology. Biorheology.

[CR11] Gomoll AH, Filardo G, Kon E (2012). SurgICAl treatment for early osteoarthritis. Part I: cartilage repair procedures. Knee Surg Sports Traumatol Arthrosc.

[CR12] Heptinstall S, White A, Henderson RA (1998). Differential effects of three radiographic contrast media on platelet aggregation and degranulation: implications for clinical practice?. Br J Haematol.

[CR13] Laadhar L (2007). Physiopathology of osteoarthritis. From normal cartilage to osteoarthritic cartilage: risk factors and inflammatory mechanisms. La Revue De Médecine Interne.

[CR14] Li X, Gabriel DA (1997). Differences between contrast media in the inhibition of platelet activation by specific platelet agonists. Acad Radiol..

[CR15] Mascarenhas R, Saltzman BM, Fortier LA, Cole BJ (2015). Role of platelet-rich plasma in articular cartilage injury and disease. J Knee Surg.

[CR16] McAlindon TE, Driban JB, Schnitzer T (2015). OARSI ClinICAl trials recommendations: design, conduct, and reporting of clinICAl trials for knee osteoarthritis. Osteoarthr Cartil.

[CR17] Nelson AE, Allen KD, Jordan JM (2014). A systematic review of recommendations and guidelines for the management of osteoarthritis: The chronic osteoarthritis management initiative of the U.S. bone and joint initiative. Semin Arthritis Rheum.

[CR18] Obermann WR, Bloem JL, Hermans J (1989). Knee arthrography: comparison of iotrolan and ioxaglate sodium meglumine. Radiology.

[CR19] Omoumi P, Mercier GA, Vande Berg BC (2009). CT arthrography, MR arthrography, PET, and scintigraphy in osteoarthritis. Radiol Clin N Am.

[CR20] Prüller F, Drexler C, Mahla E (2011). Low platelet reactivity is recovered by transfusion of stored platelets: a healthy volunteer in vivo study. J Thromb Haemost.

[CR21] Sakata R, McNary SM, Reddi AH (2015). Stimulation of the Superficial Zone Protein and LubrICAtion in the Articular Cartilage by Human Platelet-Rich Plasma. Am J Sports Med.

[CR22] Schippinger Gert, Prüller Florian, Divjak Manuela, Mahla Elisabeth, Fankhauser Florian, Rackemann Steve, Raggam Reinhard Bernd (2015). Autologous Platelet-Rich Plasma Preparations. Orthopaedic Journal of Sports Medicine.

[CR23] Seong JM, Choi NK, Park BJ (2013). Comparison of the safety of seven iodinated contrast media. J Korean Med Sci.

